# Cancer gland rupture as a potential risk factor for lymph node metastasis in early colorectal adenocarcinoma with deep submucosal invasion

**DOI:** 10.1111/his.14022

**Published:** 2020-02-24

**Authors:** Katsumi Oishi, Takashi Ito, Daisuke Sakonishi, Keisuke Uchida, Masaki Sekine, Mariko Negi, Daisuke Kobayashi, Keiko Miura, Takumi Akashi, Yoshinobu Eishi

**Affiliations:** ^1^ Department of Human Pathology Graduate School and Faculty of Medicine Tokyo Medical and Dental University Tokyo Japan; ^2^ Division of Clinical Laboratory Keio University Hospital Tokyo Japan; ^3^ Division of Surgical Pathology Tokyo Medical and Dental University Hospital Tokyo Japan

**Keywords:** algorithm, C‐shaped gland, depth of invasion, guidelines, risk assessment, T1 colorectal cancer, tumour budding, vascular invasion

## Abstract

**Aims:**

Lymph node metastasis (LNM) has not been found in more than 85% of patients with early invasive colorectal adenocarcinoma (T1‐CRAC) who undergo surgery after therapeutic endoscopy due to the risk for LNM. Better histological risk assessment for LNM of endoscopically resected T1‐CRAC is important to avoid unnecessary additional surgery.

**Methods and results:**

We evaluated cancer gland rupture (CGR), i.e. cancer glands with a discontinuous epithelial lining, at the invasive front, as a potential risk factor for LNM by histological examination of differentiated T1‐CRAC from 217 patients who underwent surgery with or without therapeutic endoscopy. CGR was represented by C‐shaped neoplastic glands with a variable inflammatory or stromal reaction, and was occasionally accompanied by mucus lake or abscess formation. CGR was observed in 168 (77%) cases, including all 20 cases with LNM, and the odds ratio of LNM was higher for CGR than for deep invasion (depth of submucosal invasion ≥1000 μm). All cases with LNM were found among 148 cases with deep invasion and positive CGR, whereas no LNM was detected in 29 cases with deep invasion and negative CGR, regardless of vascular invasion or tumour budding. In the 148 cases, LNM was detected in 18 (19%) of 93 cases with positive vascular invasion or high‐grade tumour budding, and in two (4%) of 55 cases without either.

**Conclusions:**

Our findings suggest that CGR is an easily applied and objective histological finding for predicting LNM that could be useful for assessing the risk for LNM of endoscopically resected T1‐CRAC with deep invasion.

## Introduction

Recent advancements in endoscopic instruments and techniques have increased the proportion of patients with early invasive colorectal adenocarcinoma (T1‐CRAC) initially treated using endoscopy.^1^ Subsequent surgery with lymph node dissection (salvage surgery) is recommended in Japan when one or more risk factors for lymph node metastasis (LNM) is found by histopathological examination of endoscopically resected specimens, according to the Japanese Society for Cancer of the Colon and Rectum (JSCCR) guidelines.^2^, ^3^ In the JSCCR guidelines, the risk factors for LNM include histological type of poorly differentiated adenocarcinoma, mucinous carcinoma or signet ring cell carcinoma; deep invasion (depth of submucosal invasion ≥1000 μm); positive vascular invasion (lymphatic or venous invasion); and high‐grade tumour budding (five or more foci of a single tumour cell or a cell cluster of up to four tumour cells at the invasive front).

LNM, however, is not detected in many patients with T1‐CRAC who undergo surgical resection because of the risk assessment according to the guidelines. The frequency (14%) of LNM in patients with T1‐CRAC who undergo salvage surgery after therapeutic endoscopic treatment^4^ does not differ greatly from that (≤16%) of LNM in patients with T1‐CRAC who undergo surgery without preceding endoscopic treatment.^4^, ^5^, ^6^, ^7^, ^8^, ^9^, ^10^, ^11^, ^12^ Better risk assessment for LNM of endoscopically resected specimens will help to avoid needless salvage surgery after successful endoscopic treatment of T1‐CRAC.

We found that cancer gland rupture (CGR), i.e. cancer glands with discontinuous epithelial lining, is frequent at the invasive front in many cases of T1‐CRAC in routine histological risk assessment for LNM of endoscopically removed specimens, although no attention has been paid to such a conventional histological feature in differentiated adenocarcinoma lesions. In the present study, we performed a retrospective evaluation to assess the potential usefulness of CGR as a novel predictive factor of LNM in surgical and endoscopic specimens of differentiated T1‐CRAC with or without LNM. On the basis of the results obtained in the present study, we constructed an algorithm utilising histological risk factors to assess the potential use of CGR in the risk assessment for LNM in patients with endoscopically resected T1‐CRAC with deep invasion.

## Materials and methods

### Patients

A total of 227 consecutive patients with T1‐CRAC were treated at the Tokyo Medical and Dental University Hospital between 1998 and 2015. After excluding two cases of unsuccessful endoscopic resection, three cases of poorly differentiated adenocarcinoma and five cases of mucinous carcinoma, 217 patients with differentiated T1‐CRAC, comprising 168 patients who underwent radical surgery without preceding therapeutic endoscopy, and 49 patients who underwent salvage surgery after successful therapeutic endoscopy, were retrospectively enrolled into the study (Table 1). LNM was detected in 20 (9%) of these T1‐CRAC patients. The study was approved by the ethics committee of Tokyo Medical and Dental University (Registration no. M2016‐058). The ethics committee waived the requirement for specific informed consent in accordance with the Ethical Guidelines for Clinical Studies (amended 31 July 2008) by the Ministry of Health, Labour and Welfare of Japan.

**Table 1:** Profiles of 217 patients with differentiated T1‐CRAC

Clinicopathological characteristics	
Men/women	146/71
Age, mean ± SD	66.9 ± 11.0
Tumour size, mean ± SD	21.8 ± 11.9
Therapeutic endoscopy, *n* (%)	
Present	49 (23%)
Absent	168 (77%)
Tumour type, *n* (%)	
Pedunculated	27 (12)
Non‐pedunculated	190 (88)
Histological type, *n* (%)	
Well‐differentiated	150 (69)
Moderately differentiated	58 (27)
Papillary	9 (4)
Lymph node metastasis, *n* (%)	
Present	20 (9)
Absent	197 (91)

T1‐CRAC, Early invasive colorectal adenocarcinoma; SD, Standard deviation.

### Histological Examination

CGR was evaluated by histological examination of a haematoxylin and eosin‐stained (H&E)‐stained section that included the deepest portion of each neoplastic lesion. In this study, CGR was defined as focal or partial disappearance of neoplastic epithelial cells constituting the cancer gland at the invasive front, irrespective of a coexisting inflammatory or stromal reaction, or mucus lake or abscess formation. CGR‐like features caused by artefacts due to endoscopic resection or during preparation of the histological sections were excluded. A case with at least one CGR was considered CGR‐positive regardless of the number of CGR. Conventional risk factors for LNM (depth of invasion, vascular invasion and tumour budding) were evaluated according to the JSCCR guidelines,^3^ which standardise the method for measuring the depth of invasion in both pedunculated and non‐pedunculated types of tumours.

### Statistical Analysis

κ statistics were used to assess interobserver variability in the evaluation of CGR. Fisher's exact test and multivariate logistic regression were used to evaluate the association between CGR and other risk factors. When multivariate analysis was not possible, because LNM was not found in any cases without CGR or deep invasion, only Fisher's exact test was used to evaluate the association between LNM and risk factors, including CGR. Fisher's exact test was also used to compare the LNM risk between pedunculated and non‐pedunculated cases with deep invasion. The Mann–Whitney *U*‐test was used to compare the number of CGR in cases with and without deep invasion or LNM, and to compare the depth of invasion in the low‐ and moderate‐risk cases with deep invasion. In the analysis, *P*‐values <0.05 were considered statistically significant. All analyses were carried out with the statistical package r (version 2.12.1; available from http://www.r-project.org).

## Results

### Histology of CGR

Before the analysis, CGR was independently evaluated in all the surgically resected cases by three investigators (K.O., T.I. and D.S.). Diagnostic concordance among the three observers was good (0.61–0.80; Table 2). For cases in which the evaluation differed among the three observers, the CGR status was determined by consensus following discussion among the three observers and the data were completed for the analysis.

**Table 2:** Concordance in the evaluation of CGR

	κ‐value
K.O. versus T.I.	0.76*
T.I. versus D.S.	0.66*
D.S. versus K.O.	0.68*
K.O. versus T.I. versus D.S.	0.64**

CGR, Cancer gland rupture; K.O., Katsumi Oishi; D.S., Daisuke Sakonishi; T.I., Takashi Ito.

* Cohen's κ‐value.

** Siegel's κ‐value.

**Figure 1:**
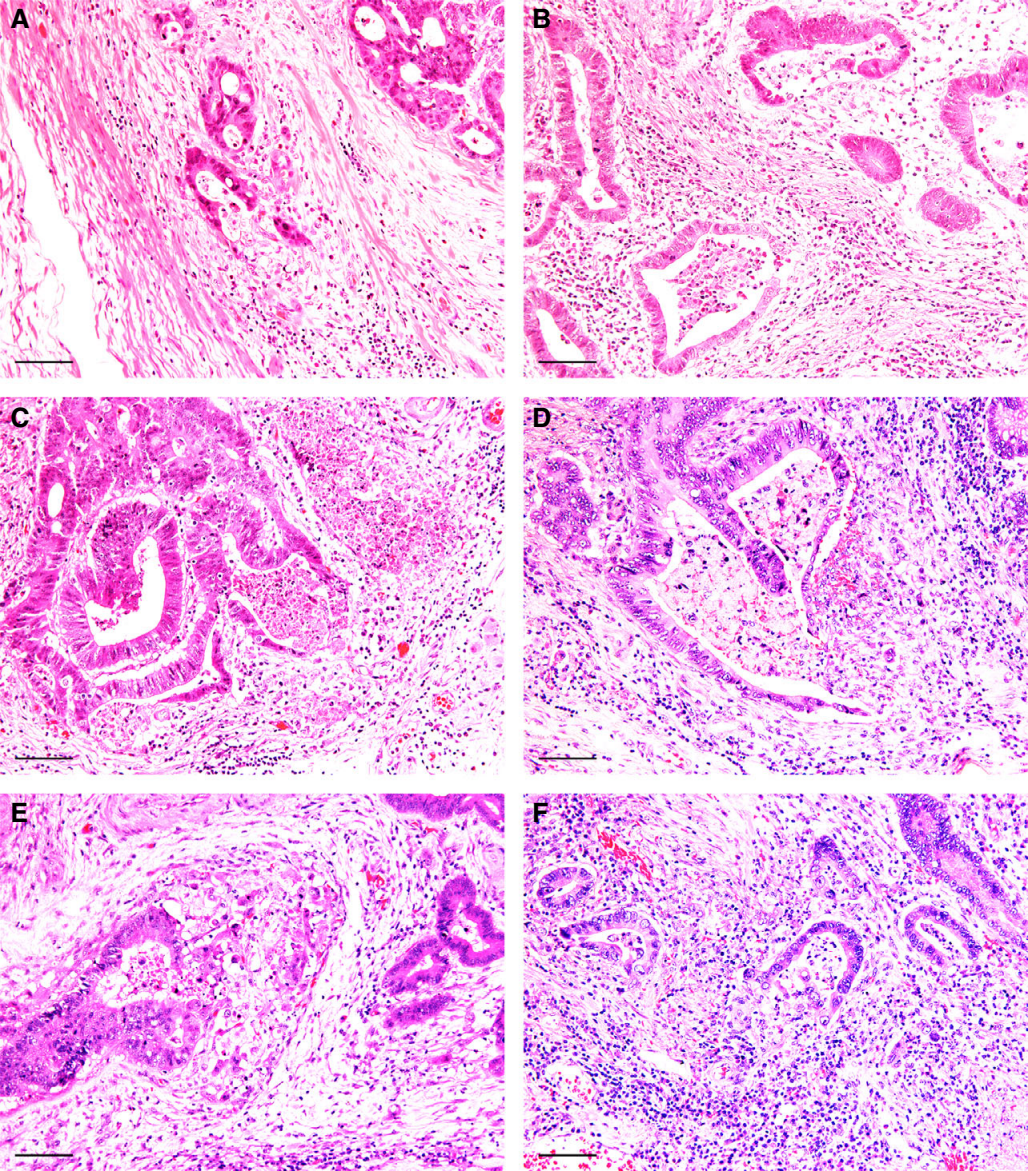
Cancer gland rupture features represented by C‐shaped glands with local reactions. C‐shaped glands with minimal local reaction (**A**) and inflammatory infiltrates (**B**) or necrosis (**C**) in the glands. Local stromal reactions observed around the C‐shaped glands (**D–F**). Scale bar: 100 µm.

**Figure 2:**
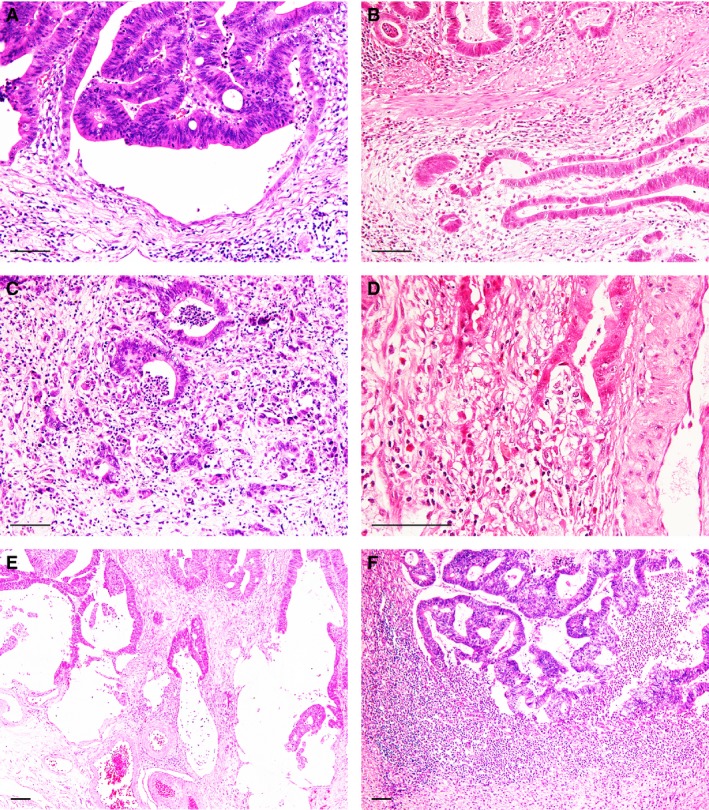
Histological features in relation to the causes of cancer gland rupture (CGR). Gradual flattening of the cancer cell lining (**A**), discrete foci of cancer cells from the C‐shaped glands (**B**), CGR with poorly differentiated clusters or tumour budding (**C**) and transitional appearance between glandular and stromal cancer cells (**D**). CGR with mucus lake formation (**E**) or abscess formation (**F**). Scale bar: 100 µm.

CGR was identified in 168 (77%) of 217 T1‐CRAC cases. The typical feature of C‐shaped cancer glands was observed in 139 (83%) of the 168 CGR‐positive lesions, showing variable inflammatory infiltrates or necrosis in the glands and inflammatory or stromal reaction around the glands (Figure 1). Other features indicative of CGR included flattening or dissociation of the cancer cell lining, coexistence of poorly differentiated clusters and tumour budding, and mucus lake or abscess formation at the invasive front (Figure 2). CGR with mucus lake or abscess formation was observed in 17 (10%) and 12 (7%) of the lesions, respectively, including five lesions with a typical C‐shaped pattern in another area. Except for massive abscess formation, CGR was focal in most cases and the median number of CGR was 2 (25th and 75th percentiles: 1 and 5, respectively).

### CGR and Other Risk Factors

The association between CGR and other risk factors for LNM was evaluated (Table 3). Univariate analysis revealed that CGR was not associated with lymphatic invasion and tumour budding, but was associated with depth of invasion (*P* < 0.001) and venous invasion (*P* = 0.021). Multivariate analysis for CGR of these risk factors, however, revealed only depth of invasion as an independent factor in the association (*P* < 0.001). CGR was not detected in 29 (16%) of the 177 lesions with deep invasion. The median number of CGR in cases with deep invasion was higher than that in cases without deep invasion (3.0 versus 0.5, *P* < 0.0001).

**Table 3:** Association between CGR and other risk factors for LNM

	*n* (%)	Number of patients		*P*‐value*
		With CGR (*n* = 168)	Without CGR (*n* = 49)	
Depth of SM invasion				
≥1000 µm	177 (82)	148	29	<0.001
<1000 µm	40 (18)	20	20	
Lymphatic invasion				
Positive	27 (12)	19	8	NS
Negative	190 (88)	149	41	
Venous invasion				
Positive	89 (41)	76	13	0.021
Negative	128 (59)	92	36	
Tumour budding				
High‐grade	66 (30)	57	9	NS
Low‐grade	151 (70)	111	40	

CGR, Cancer gland rupture; LNM, Lymph node metastasis; SM, Submucosal; NS, Not significant.

* Fisher's exact test. Depth of SM invasion was an independent factor (*P* < 0.001) in the association with CGR by multivariate logistic regression analysis.

### CGR and LNM

To evaluate the potential value of CGR as a predictor of LNM, we examined CGR and other risk factors for LNM (Table 4). All 20 cases with LNM were positive for CGR. Among the 197 cases without LNM, 49 (25%) were negative for CGR. The associations between LNM and each risk factor, including CGR and LNM, were all statistically significant. The odds ratio for LNM was highest in CGR (13.7) and second highest (10.5) in depth of invasion, followed by lymphatic invasion (3.6), tumour budding (3.2) and venous invasion (3.0). Sensitivity to predict LNM was high (100%) for both CGR and depth of invasion, whereas the specificity of these risk factors was low (25% and 20%, respectively). Conversely, lymphatic invasion had high specificity (89%) and low sensitivity (30%). The sensitivity and specificity of venous invasion or tumour budding were intermediate: 65% and 61% and 55% and 72%, respectively. The median number of CGR in cases with LNM was higher than that in cases without LNM (3.5 versus 2.0, *P* = 0.025).

**Table 4:** Odds ratio, sensitivity, and specificity of CGR and other risk factors for LNM

	Number of patients		Odds ratio	*P*‐value*	Sensitivity (%)	Specificity (%)
	With LNM (*n* = 20)	Without LNM (*n* = 197)				
CGR						
Positive	20	148	13.7	0.009	100	25
Negative	0	49				
Depth of SM invasion						
≥1000 µm	20	157	10.5	0.029	100	20
<1000 µm	0	40				
Lymphatic invasion						
Positive	6	21	3.6	0.024	30	89
Negative	14	176				
Venous invasion						
Positive	13	76	3.0	0.031	65	61
Negative	7	121				
Tumour budding						
High‐grade	11	55	3.2	0.020	55	72
Low‐grade	9	142				

CGR, Cancer gland rupture; LNM, Lymph node metastasis; SM, Submucosal.

* Fisher's exact test. Multivariate analysis was not possible because there were no cases with LNM that were CGR‐negative or had SM invasion depth <1000 µm.

### Algorithm Construction

On the basis of the high sensitivity of CGR and deep invasion as risk factors for LNM, we classified the cases of T1‐CRAC into four groups: CGR‐positive cases with or without deep invasion and CGR‐negative cases with or without deep invasion. In these four groups, the status of LNM and the other three risk factors was evaluated (Table 5). LNM was not detected in any of the 40 cases without deep invasion, regardless of CGR or other risk factors. In the 177 cases with deep invasion LNM was not detected in any of the 29 (16%) CGR‐negative cases regardless of the other three risk factors, whereas LNM was detected in 20 (14%) of the 148 cases with both CGR and deep invasion.

**Table 5:** Status of LNM and other risk factors in the four groups classified on the basis of depth of invasion and CGR status

	Number of cases with SM invasion			
	≥1000 µm		<1000 µm	
	With CGR (*n* = 148)	Without CGR (*n* = 29)	With CGR (*n* = 20)	Without CGR (*n* = 20)
Lymphatic invasion				
Positive	17	5	2	3
Negative	131	24	18	17
Venous invasion				
Positive	71	9	5	4
Negative	77	20	15	16
Tumour budding				
High‐grade	51	7	6	2
Low‐grade	97	22	14	18
LNM				
Present	20	0	0	0
Absent	128	29	20	20

CGR, Cancer gland rupture; LNM, Lymph node metastasis; SM, Submucosal.

### Risk Classification

**Figure 3:**
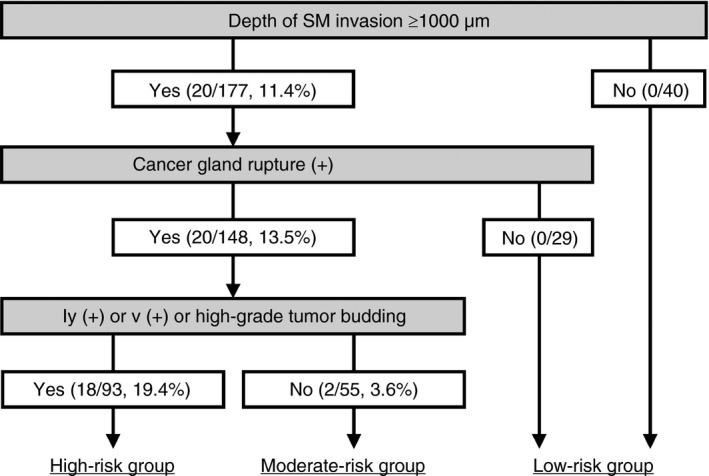
Risk assessment algorithm for lymph node metastasis (LNM) in early invasive colorectal adenocarcinoma (T1‐CRAC) patients. Proportion of the patients with LNM is shown by the number (%) per total number of patients in each white column with yes (presence) or no (absence) of the risk factor(s) mentioned in the upper grey column. The 177 T1‐CRAC patients with deep invasion were classified as 93 (53%) high‐risk, 55 (31%) moderate‐risk and 29 (16%) low‐risk for LNM using cancer gland rupture as an entry risk factor in the algorithm.

Based on the results obtained, we constructed a T1‐CRAC risk classification system including CGR as one of the risk factors for LNM (Figure 3). Cases without deep invasion were all classified in the low‐risk group irrespective of the status of the other risk factors, including CGR. The cases with deep invasion were also classified into the low‐risk group when they were negative for CGR, irrespective of the status of the other risk factors. Cases with both deep invasion and CGR were classified in the high‐risk group when they exhibited either vascular invasion or tumour budding; otherwise, they were classified into the moderate‐risk group. Using this algorithm to assign the level of risk, LNM was detected in none (0%) of the 69 low‐, two (4%) of the 55 moderate‐ and 18 (19%) of the 93 high‐risk patients.

It should be noted that in the 49 cases resected endoscopically, LNM was detected in none (0%) of the 18 low‐ and 16 moderate‐risk patients and in four (27%) of the 15 high‐risk patients. No difference was detected in the proportion of low‐, moderate‐ and high‐risk patients by the algorithm between settings with or without the 49 cases that were resected endoscopically (32% versus 31%, 25% versus 23% and 43% versus 46%, respectively).

The median depth of invasion in the cases with deep invasion was higher in the 55 moderate‐risk cases than in the 29 low‐risk cases (3000 versus 2000 μm, respectively, *P* = 0.0096). No significant difference was detected in the risk for LNM according to the algorithm between pedunculated and non‐pedunculated cases with deep invasion: LNM was found in two (17%) of 12 pedunculated and 18 (11%) of 165 non‐pedunculated cases with deep invasion (*P* = 0.63).

### Benefit of the Algorithm

**Figure 4:**
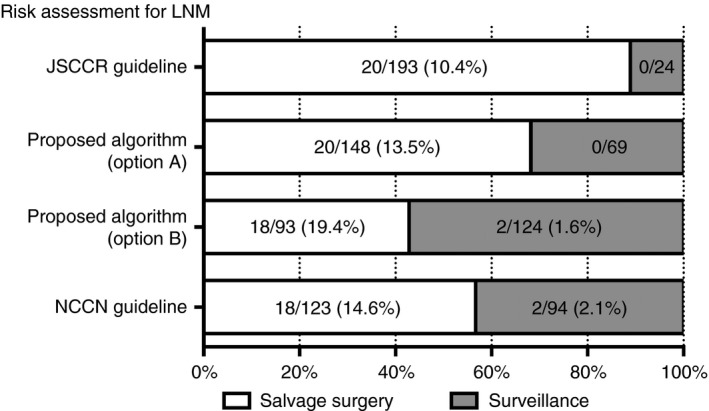
Assumed benefit of the risk assessment algorithm for lymph node metastasis (LNM) in practical use. Proportions of 217 early invasive colorectal adenocarcinoma (T1‐CRAC) patients who would be operated on (salvage surgery) or followed‐up (surveillance) after successful therapeutic endoscopy are shown in the white or grey column with the risk for LNM, respectively, according to the criteria of the current Japanese Society for Cancer of the Colon and Rectum (JSCCR), National Comprehensive Cancer Network (NCCN) guidelines and the algorithm proposed in the present study. The results are shown for two options: when the salvage operation is performed for both high‐ and moderate‐risk patients (option A) or for only high‐risk patients (option B). The risk for LNM is indicated by the proportion (%) of patients with LNM per total number of patients in each group with or without salvage surgery.

The benefit of the algorithm was evaluated in comparison with the current JSCCR guidelines (Figure 4). The analysis addressed the following question: if successful endoscopic resection was performed for all 217 lesions evaluated in the present study, how many patients would require salvage surgery or could be followed‐up (surveillance) without surgery according to the JSCCR guidelines and the proposed algorithm? We report the results for cases who underwent salvage surgery among both high‐ and moderate‐risk patients (option A) or among only high‐risk patients (option B). Compared with the JSCCR guidelines, when applying the algorithm to both high‐ and moderate‐risk patients (option A), the proportion of patients requiring surgery decreased from 89% to 68% and the frequency of metastasis in the dissected lymph nodes increased from 10% to 14%. When applying the algorithm to only high‐risk patients (option B), the proportion of patients requiring surgery further decreased to 43% and the frequency of LNM increased (up to 19%) in those patients, although LNM is found in just a few of the follow‐up patients. Additionally, compared with the National Comprehensive Cancer Network (NCCN) guidelines,^13^, ^14^ which do not include depth of invasion but do include vascular invasion and tumour budding as unfavourable risk factors for LNM, applying option B decreased the proportion of patients requiring surgery from 57% to 43%, but did not change the frequency of metastasis in the follow‐up patients (2% in the NCCN guidelines and option B, respectively).

## Discussion

This is the first study, to our knowledge, that describes the relation between CGR and LNM in T1‐CRAC patients. Evaluation of CGR in H&E‐stained sections was easy in many cases, using a low‐power view and objective criteria, and with good diagnostic concordance among observers. CGR was represented by C‐shaped cancer glands with a variable inflammatory or stromal reaction and was occasionally accompanied by a mucus lake or abscess formation at the invasive front. CGR was detected in many cases of T1‐CRAC and significantly correlated with both LNM and depth of invasion, with high sensitivity and low specificity for LNM compared with other risk factors, such as vascular invasion and tumour budding. For the potential use of CGR in the risk assessment for LNM of T1‐CRAC, we constructed an algorithm of histological risk factors, including CGR, and classified the patients with T1‐CRAC into three LNM risk groups according to the results obtained in the study. The algorithm proposed here for the risk assessment for LNM of differentiated T1‐CRAC will help to increase the proportion of T1‐CRAC patients who can be followed‐up without salvage surgery after successful therapeutic endoscopy.

In western guidelines for the treatment of colorectal cancer,^13^, ^14^, ^15^ patients with tumours having at least one of the histological risk factors (unfavourable histological features) are recommended to receive additional surgery. Also, in the Japanese guidelines,^2^, ^3^, ^16^ when one or more risk factors for LNM are found in endoscopically resected specimens, patients with T1‐CRAC are recommended to undergo salvage surgery, even after successful endoscopic treatment. These histological risk assessments can lead to recommendations for salvage surgery in most patients with LNM, but such a simultaneous parallel processing of risk factors with different sensitivities and specificities for LNM has led to needless surgery in many T1‐CRAC patients without LNM.^8^, ^9^, ^11^, ^12^

To avoid needless salvage surgery, an appropriate algorithm is needed that utilises the risk factors and incorporates the sensitivity and specificity of the risk factors for LNM. For constructing such an algorithm, we need to select risk factors for LNM with high enough sensitivity (despite low specificity) as the major entry risk factors. For this purpose, Kawachi *et al.* recently proposed a three‐tier classification of the risk for LNM based on depth of invasion and tumour budding, where LNM was found in 2% of low‐, 9% of intermediate‐ and 29% of high‐risk patients.^17^ In the present study, both CGR and deep invasion were risk factors with high sensitivity (100%) for LNM. Dichotomising all cases according to the presence or absence of CGR or by the depth of invasion (i.e. <1000 µm or ≥1000 µm) was essential for maintaining high sensitivity. We used these two histological findings as the major entry risk factors in the proposed algorithm.

Using the novel risk factor of CGR in the algorithm allowed us to classify the 177 T1‐CRAC patients with deep invasion into the three LNM risk groups, where LNM was detected in 0% of low‐, 4% of moderate‐ and 19% of high‐risk patients. The 84 (47%) patients in the low‐ and moderate‐risk groups will benefit from the proposed algorithm, as they may otherwise undergo salvage surgery due to deep tumour invasion according to the current Japanese guidelines. How moderate‐risk patients should be treated (options A or B) under Japanese guidelines, which aim to guarantee no risk (0%) for LNM during surveillance after therapeutic endoscopy, may depend upon each individual patient's clinical characteristics, such as age and presence or absence of complications.^13^ Even when compared with current Western guidelines, the algorithm (option B) would help to decrease the proportion of patients requiring salvage surgery.

The total number of T1‐CRAC patients used for the analysis may be not large enough to advocate incorporation of CGR into the diagnostic criteria or to conclusively determine that the algorithm is useful for assessing LNM risk. Further studies are needed, including evaluation of CGR in other study groups and confirmation of the current results in larger cohorts. T1‐CRAC with deep invasion but no CGR detected may be an especially important issue for future studies, because these patients can be followed‐up without any risk for LNM regardless of vascular invasion or tumour budding. To increase the overall study population, both T1‐CRAC cases with or without preceding therapeutic endoscopy were included in the present study. In both settings, no difference was found in the proportions of the three‐tier risk groups categorised by the current algorithm.

The present study has some limitations. Although we found no LNM in the 40 T1‐CRAC cases without deep invasion, previous studies found LNM in 0–2.9% of T1‐CRAC cases without deep invasion (<1000 μm) irrespective of other risk factors.^8^, ^9^, ^11^, ^12^, ^17^ The risk of LNM in patients with T1‐CRAC without deep invasion seems to be generally low, and these patients may be carefully followed without additional surgery.^17^ The limitations in the present study should be addressed by future studies, because even if the risk is not 0% according to the current algorithm, patients should be able to choose further management on the basis of the most accurate information possible regarding their risk for LNM. Another limitation is that the CGR risk factor cannot be used for other histological types, including poorly differentiated adenocarcinoma, mucinous carcinoma and signet ring cell carcinoma. For practical use, however, the proportion of T1‐CRAC patients with these unfavourable histological types is small (3.5% in the present study), and these patients may be better classified into the high‐risk group, regardless of any other risk factors for LNM.

Depth of invasion was the only independent risk factor associated with CGR. Discrimination of the cases with deep invasion between low‐ and moderate‐risk groups by CGR was also associated with depth of invasion. CGR may merely be a surrogate marker of depth of invasion, while the cause or mechanism of CGR remains unknown. The CGR features observed in the present study suggest that CGR may be a histological finding related to the epithelial–mesenchymal transition (EMT), a cellular phenomenon in which epithelial cells lose their cell–cell adhesion and gain the migratory and invasive traits typical of mesenchymal cells.^18^, ^19^ Recent studies have demonstrated that tumour budding is closely associated with EMT.^20^, ^21^, ^22^, ^23^ Indeed, CGR is observed predominantly at the invasive front and is accompanied in some cases by poorly differentiated clusters^24^, ^25^, ^26^ or tumour budding. Alternatively, the low specificity of CGR as a predictor of LNM suggests that CGR reflects not only EMT, but also a secondary phenomenon subsequent to a non‐specific tissue reaction, such as focal cancer cell necrosis, inflammatory cell infiltration and rupture of cancer glands by mucus retention. Verification of the association between CGR and EMT requires future expression analyses of EMT‐associated proteins^18^ in cancer glands with CGR.

## Conflict of interest

The authors declare no conflicts of interest associated with this manuscript.
